# Biocontrol of phytopathogenic fungi by *Bacillus* spp.: Strain screening, regulatory mechanisms, and field application

**DOI:** 10.3934/microbiol.2026025

**Published:** 2026-08-28

**Authors:** Feng Wang, Shuang Zhang, Ling Xu, Jingya Qian, Bin Zou, Shuhao Huo, Anzhou Ma, Guoqiang Zhuang

**Affiliations:** 1 School of Food and Biological Engineering, Jiangsu University, Zhenjiang 212013, China; 2 Research Center for Eco-Environmental Sciences, Chinese Academy of Sciences, Beijing 100085, China; 3 University of Chinese Academy of Sciences, Beijing 100049, China

**Keywords:** *Bacillus* spp., biological control, phytopathogenic fungi, lipopeptides, induced systemic resistance, rhizosphere microbiome, sustainable agriculture

## Abstract

*Bacillus* species, which are among the most extensively studied and widely used rhizobacteria, have demonstrated potent antagonistic activity against a broad spectrum of phytopathogenic fungi, making them ideal microorganisms for biological control. Phytopathogenic fungi cause severe preharvest and postharvest losses in global crop production, significantly reducing yield and quality. The prolonged and heavy reliance on traditional chemical fungicides has resulted in environmental pollution and poses risks to human health. In this context, *Bacillus*-based biological control has emerged as an environmentally friendly, cost-effective, and sustainable alternative strategy. In this review, we provide a comprehensive overview of the screening of *Bacillus* strains for the biocontrol of phytopathogenic fungi. The possible mechanisms, including the production of a wide array of antifungal metabolites, the induction of systemic resistance in host plants, and indirect suppression through competition for nutrients and space, were also evaluated. Furthermore, the effects of multistrain synergistic consortia and the development of multifunctional *Bacillus* strains on biocontrol efficacy are discussed, and research advances in the application of *Bacillus* spp. in the control of soilborne diseases and phytopathogenic fungi are summarized. Finally, this review provides suggestions for the development of efficient *Bacillus*-based biocontrol agents, thereby promoting sustainable agricultural development.

## 1. Introduction

*Bacillus* species are gram-positive bacteria that are widely distributed in soil and plant-associated microenvironments [1],[2]. As *Bacillus* species produce a variety of enzymes, antibiotics, and metabolites, which can inhibit pathogens and have broad-spectrum antibacterial activity, they have become a promising biological control agent in sustainable agriculture [3],[4]. Some *Bacillus* species have been identified as plant growth-promoting rhizobacteria (PGPR), which can directly promote plant growth through nitrogen fixation and phosphorus dissolution as well as promote the production of plant hormones. Moreover, they produce antibiotics, lyases, and siderophores and have antagonistic effects on a variety of plant pathogens [5],[6]. Therefore, *Bacillus* is considered to be among the most promising chassis microorganisms for the development of microbial fungicides and biofertilizers. Moreover, species such as *Bacillus subtilis* and *B. thuringiensis* have been applied to disease management [7]–[10].

Pathogenic plant fungi cause 70% to 80% of plant diseases, posing a persistent threat to global agricultural productivity, resulting in severe yield losses and deterioration of major crop quality [11],[12]. Examples include wheat rust caused by *Puccinia* spp. [13], wilts and head blight of crops caused by *Fusarium* spp. [14], and grey mould of fruits and vegetables caused by *Botrytis cinerea* [15]. Interventions against these fungal diseases rely mainly on the breeding of resistant varieties, crop rotation, optimal planting density, and the application of chemical fungicides [16]. However, the breeding cycle for resistant varieties is lengthy, and the rapid genetic variation of pathogenic fungal populations often leads to the breakdown of resistance within a few years of deployment [17]. The primary measure against plant pathogenic fungi is the application of synthetic chemical pesticides. However, the continuous and intensive use of synthetic chemical pesticides in agriculture may exacerbate environmental pollution [18],[19], and their residues are considered carcinogenic and pose risks to human health [20]. Moreover, the prolonged use of synthetic chemical pesticides has led to increased resistance in fungal pathogens, limiting their efficacy [21],[22]. Consequently, the search for new methods to control these plant pathogenic fungi and manage fungal pathogens in an environmentally friendly manner has stimulated the research and development of novel control agents [23],[24]. Furthermore, with increasing health awareness and the advancement of sustainable agriculture, research on biocontrol agents is becoming increasingly recognized as an effective approach to mitigate the impact of fungal diseases [25]–[27].

In this context, *Bacillus* species demonstrate unique advantages as biocontrol agents against plant pathogenic fungi [28],[29]. The genus *Bacillus* has been widely applied in the control of plant pathogenic fungi because of its distinctive ability to form spores; the unique spore structure enables them to grow under adverse conditions. When these spores are applied to plants as dormant cells, they germinate into metabolically active cells. After germination, the bacteria are attracted by chemotaxis, subsequently undergo root colonization, and exert their effects [5]. Kamath et al. [30] reported that *Bacillus* species could increase root colonization, promote plant growth, and significantly inhibit the growth of plant pathogenic fungi. *Bacillus* species control plant pathogenic fungi through the secretion of volatile organic compounds, utilization of siderophores, and production of hydrolytic enzymes. Figueiredo et al. [31] reported that *Bacillus velezensis* harbors antifungal genes responsible for the synthesis of volatile organic compounds, biofilm formation, and hydrolytic enzymes, which can significantly inhibit fungal growth and increase crop yield. The synergy of multiple mechanisms not only improves biocontrol efficacy but also has growth-promoting effects. Some *Bacillus* species also achieve control by competing for nutrients and ecological niches. Zhu et al. [32] reported that *B. velezensis* BV07 could colonize the surface of citrus wounds, effectively competing for space and nutrients by consuming available resources and thereby inhibiting the growth and infection of pathogens. In nature, the antagonistic effect of *Bacillus* against plant pathogenic fungi is not dependent on a single strain but often involves synergistic interactions with other strains. Zhang et al. [33] showed that treatment with a mixed *Bacillus* strain consortium altered the community composition of the rhizosphere microbiome, significantly enhancing plant resistance to plant pathogenic fungi.

A review of the related literature from the past five years revealed that, while *Bacillus* species have been extensively documented as important biocontrol agents, most reviews have concentrated on their antimicrobial metabolites [34]–[36] or have been limited to specific crop–pathogen systems [37]–[39]. Given the vast body of research data on *Bacillus* and plant pathogenic fungi, a single review cannot encompass all aspects; thus, an integrative perspective spanning from laboratory strain screening to field application is required. In this review, we aimed to outline the pathway of strain screening: mechanistic diversity—microbial consortium synergy—field application, thereby providing a structured knowledge map, and to place particular emphasis on the ecological synergy of synthetic microbial consortia and the application of *Bacillus* species in extreme agricultural environments such as high temperature, drought, and saline-alkali conditions.

## 2. Screening of biocontrol *Bacillus* spp.

The genus *Bacillus* comprises aerobic or facultatively anaerobic, gram-positive, spore-forming rods. Owing to the high resistance of their spores to extreme environments, they are widely distributed in nature. In agriculture, biocontrol *Bacillus* strains are often screened from soil in which healthy plants grow or from plant tissues, and they are widely used as biofertilizers and biopesticides, playing important roles in disease control, stress tolerance, nutrient uptake, and yield improvement of plants [40]. In certain screening processes, rational design can also be employed to obtain the desired strains. As shown in Table 1, we have summarized the screening of biocontrol *Bacillus* strains over the past five years.

**Table 1.** Screening of biocontrol *Bacillus* spp.

Strain ID/Name	Isolation source	Target pathogen	Screening method	Biocontrol mechanism	Ref.
*Bacillus subtilis* Z-14	Wheat rhizosphere soil	*Gaumannomyces graminis var. tritici*	Dual culture plate assay	fengycins, iturin A	[41]
*B. amyloliquefaciens*	Wheat rhizosphere soil	*Fusarium equiseti*	Dual culture plate assay	iturins, fengycins, surfactins	[42]
*B. velezensis* T149-19	Sweet potato root endophyte	*Plenodomus destruens*	Whole‑genome sequencing	mycosubtilins, bacillaenes, macrolactins, fengycins	[43]
*B. velezensis* Ag75	Maize rhizosphere soil	*Rhizoctonia solani*	Whole‑genome sequencing	Surfactins, fengycins, bacillibactins	[44]
*B. velezensis* S41L	Asymptomatic pear tree rhizosphere soil	*Rosellinia necatrix*	Cell-Free Supernatantt	Volatile organic compounds, lipopeptides	[45]
*Bacillus halotolerans* NYG5	Agricultural field soil	*M. phaseolina*	Two‑compartment plate	2,3‑hexanedione	[46]
*B. subtilis* KU21	Rosemary root endophyte	*F. oxysporum*, *F. graminearum*, *R. solani*	Dual culture assay	IAA, siderophores, HCN	[47]
*Bacillus licheniformis* AD70	Cucumber rhizosphere soil	*F. oxysporum*, *Aspergillus niger*	Selenium tolerance screening	SeNPs	[48]
*B. velezensis* QY-4	Healthy strawberry plant and rhizosphere soil	*Neopestalotiopsis clavispora*	Dual culture assay, 16S rRNA	Volatile organic compounds	[49]
*Bacillus subtilis* YLX6	Blue honeysuckle healthy leaves	*Alternaria alternata*	Dual culture plate assay	Surfactins, iturin A	[50]

Isolating and screening *Bacillus* strains with antifungal activity from natural environments is the most traditional and effective approach for developing biocontrol agents. Soil, the plant rhizosphere, endophytic tissues, and extreme environments serve as important sources of *Bacillus* [42],[51]–[53]. Most screening methods are based on well-known tests of the interaction modes between microbial strains and plants or pathogens. Moreover, biocontrol strains exhibiting direct antagonistic activity are screened using solid or liquid media. Researchers often isolate candidate strains from target plants and evaluate their antagonistic ability against phytopathogenic isolates. For example, Almasoudi et al. [54] obtained ten bacterial isolates from the tomato rhizosphere and tested them against the pathogenic isolate *Fusarium oxysporum* f. sp. *lycopersici* (KAUK7), which was isolated from tomato plants. A conventional screening workflow typically includes sample collection, serial dilution and plating on nutrient agar or selective media, preliminary identification, and then primary screening for antagonistic strains through a dual culture assay. The inhibition rate is calculated by measuring the fungal growth radius [55],[56]. During the secondary screening stage, the broad-spectrum antifungal activity of the strains is further evaluated, and their ability to produce inhibitory volatile organic compounds as well as antifungal substances such as hydrolytic enzymes, siderophores, and lipopeptides is assessed [57],[58]. To screen strains that induce induced systemic resistance (ISR), in planta assays are required. Some researchers have also tested strains for salt, drought, or heat tolerance to select candidates suitable for harsh agricultural environments [59]. Santhosh et al. [58] isolated an endophytic bacterium from eggplant roots that was able to colonize the root system. The strain was found to reduce H₂O₂ accumulation and increase the activity of defence enzymes such as SOD, POD, PPO, and PAL, demonstrating not only biocontrol efficacy but also multiple plant growth-promoting activities. After 16S rDNA sequencing, it was identified as *B. velezensis*.

Furthermore, screening strains through microbiome analysis has been considered an effective strategy. Studies based on microbiome analysis have revealed that specific microorganisms can confer resistance to pathogens in crops [60],[61]. For example, Zhuang et al. [62] collected surface microorganisms every six days and rapidly obtained beneficial microorganisms by monitoring the dynamics of the microbiome; they also found that, compared with single-strain treatments, tomato plants treated with different ratios of *B. velezensis* B1 and *M. caribbica* Y9 as a composite inoculant showed significantly improved control efficacy against pathogens, indicating that composite inoculants can provide better plant protection than single strains [63].

In summary, based on strain activity, genetic characteristics, and their interactions with other microorganisms or plants, candidate Bacillus strains, either individually or in combination, can be selected for the development of microbial agents. Furthermore, attention should be given to their safety, environmental risks, and ecological behaviors when selecting microorganisms to promote the sustainable development of agriculture.

## 3. *Bacillus*-mediated biocontrol mechanisms of phytopathogenic fungi

*Bacillus* species employ a range of mechanisms to suppress plant pathogens and pests, rendering them effective biocontrol agents in cropping systems [64]. These mechanisms can be broadly classified into three categories: First, they produce a variety of secondary metabolites, whose biosynthesis accounts for 5–8% of the total genome, that mediate antimicrobial activity [65], such as lipopeptides, volatile organic compounds, and hydrolytic enzymes; second, they efficiently compete for iron ions and ecological niches in the environment, thereby reducing the living space of pathogens; and third, they act as biocontrol elicitors to induce systemic resistance in host plants. These three mechanisms often act synergistically, collectively forming the biocontrol barrier of *Bacillus* [66]. In this section, the biocontrol mechanisms employed by *Bacillus* against plant pathogenic fungi based on these three categories are introduced.

### 3.1. Production of antimicrobial secondary metabolites

#### 3.1.1. Lipopeptides

Lipopeptides are among the most extensively studied and mechanistically well-defined secondary metabolites produced by *Bacillus* species and play a central role in the biological control of plant diseases. They are amphiphilic molecules composed of a hydrophobic fatty acid chain linked to a hydrophilic cyclic peptide moiety via lactone or lactam bonds. On the basis of their cyclic peptide structure, they can be divided into three families: The surfactin family [67], the fengycin family, and the iturin family [68]. Although members of different families vary in peptide chain length (mostly 7–10 amino acids) and fatty acid chain carbon number (C13–C19), their common target is the cell membrane of pathogenic fungi. As shown in Figure 1, the typical mode of action involves the insertion of their amphiphilic structure into the fungal plasma membrane, where they interact with membrane lipids and cause damage to the cytoplasmic membrane, leading to intracellular ion leakage, cytoplasmic efflux, and cell death [69]. For instance, bacillomycin produced by *Bacillus amyloliquefaciens* is an antifungal lipopeptide that serves as a biocontrol agent with favourable environmental adaptability and a membrane-targeting mechanism. Liu et al. [70] exploited bacillomycin to disrupt the cell membrane integrity of *Fusarium graminearum*, providing a direction for the control of maize ear rot or stalk rot.

Surfactins have immense potential in agriculture, food processing, and pharmaceuticals and exhibit broad-spectrum antibacterial, antiviral, and antitumor activities, which are attributable to their structural stability, strong surface activity, and intrinsic antibiotic properties [71]–[73]. Surfactins belong to the cyclic lipopeptide family and are a representative biosurfactant. They are synthesized by nonribosomal peptide synthetases (NRPSs) and exported extracellularly, consisting of a cyclic heptapeptide linked to a variable-length β-hydroxy fatty acid chain containing 11–18 carbon atoms [74]. As amphiphilic molecules, surfactins insert into lipid bilayers via hydrophobic interactions, causing dehydration of phospholipid polar head groups, decreasing bilayer stability, and disrupting membrane barrier function and permeability through mechanisms such as channel formation. Moreover, their acidic residues can chelate monovalent and divalent cations; divalent cations neutralize the charge and form 1:1 complexes, promoting deeper insertion of surfactin into the membrane and thereby enhancing its antimicrobial activity [75]. However, owing to the low yield of surfactins in natural strains, Liu et al. [76] optimized the fermentation conditions of *B. velezensis* BN through single-factor experiments and response surface methodology, resulting in a 5.94-fold increase in surfactin production and a significant increase in the antifungal activity of the strain against pathogenic fungi.

**Figure 1.**
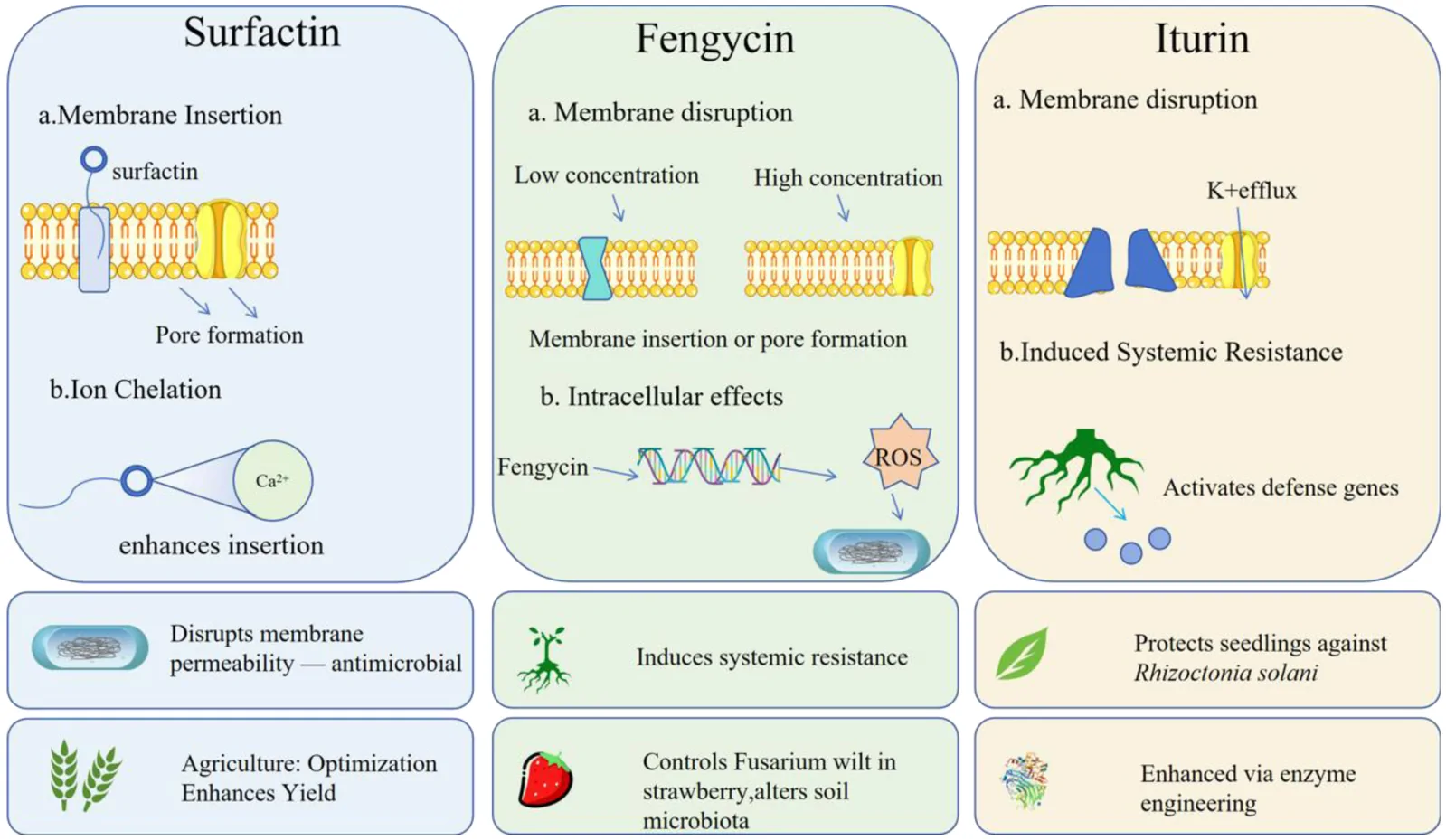
Surfactin forms membrane pores and chelates metal ions to disrupt fungal cell membranes; Fengycin damages fungal membranes, induces intracellular oxidative apoptosis, and elicits plant systemic resistance; and Iturin disrupts fungal potassium ion homeostasis, causing cell death, while activating plant defense genes.

Fengycins disrupt cell wall integrity by inducing the release of cell wall hydrolytic enzymes from fungi and downregulating the expression of key genes involved in cell wall synthesis [77]. At low concentrations, they insert into the membrane and cause local perturbation, whereas at high concentrations, they form aggregates or pores, leading to increased membrane permeability, leakage of cellular contents, and cell death [78]. Furthermore, fengycins can enter cells, bind to DNA, and interfere with gene expression, inhibiting toxin synthesis and energy metabolism while inducing the production of reactive oxygen species and activating the mitochondrial apoptosis pathway in fungi. In plants, they elicit systemic resistance through the jasmonic acid/salicylic acid signaling pathway, thereby strengthening the host's defence against pathogens [79],[80]. Chen et al. [81] demonstrated that fengycins produced by *B. subtilis* could directly inhibit the growth of *Fusarium* to control strawberry wilt. The investigators also reported that fengycin treatment significantly altered the soil microbial community structure, reduced the relative abundance of pathogenic fungi, and increased the abundance of beneficial microorganisms.

The iturin biosynthesis-related gene cluster typically comprises genes such as *ituA*, *ituB*, *ituC*, and *ituD*, which encompass functions responsible for amino acid activation, transport, and peptide chain assembly. These genes direct the synthesis of iturin, endowing it with antifungal and other biological activities [82]. Iturin exerts broad-spectrum antifungal activity by directly targeting the fungal cell membrane, disrupting its integrity, and activating voltage-gated potassium channels, which causes massive potassium efflux, disrupts intracellular ion homeostasis, and induces fungal cell death [83]. Concurrently, iturin acts as an elicitor to induce systemic resistance (ISR) in plants, activating the expression of defence-related genes and enhancing plant immunity against pathogens [3]. Kumar et al. [84] reported that an endophytic bacterium from sorghum–sudangrass seeds could produce iturin A, effectively protecting seedlings against *Rhizoctonia solani* infection and demonstrating its potential as a biocontrol agent. To expand the application of iturin A, Zhang et al. [85] enhanced iturin A enzymatic activity through gene cluster regulation and rational enzyme design, significantly improving its antifungal efficacy against many molds.

#### 3.1.2. Volatile organic compounds

Volatile organic compounds (VOCs) can directly inhibit hyphal growth, sporulation, and spore germination in pathogenic fungi and downregulate the expression of genes related to fungal chitin synthase, ergosterol biosynthesis, cell wall-degrading enzymes, and energy metabolism at the transcriptional level, thereby attenuating fungal pathogenicity [86]. Some VOCs also induce the accumulation of reactive oxygen species in fungal conidia, causing DNA and cell membrane damage [87]. Moreover, VOCs released by *Bacillus* species, such as acetoin and 2,3-butanediol, can trigger ISR in plants, upregulating the expression of defence-related genes through the ethylene, jasmonic acid, and salicylic acid signaling pathways and indirectly enhancing plant resistance to fungal pathogens [35]. He et al. [88] reported that VOCs produced by *B. velezensis*, predominantly isovaleric acid, could effectively inhibit the hyphal growth and spore germination of *Alternaria alternata* by disrupting cell wall and cell membrane integrity, impairing mitochondrial function, reducing pyruvate content, and weakening antioxidant capacity, thereby achieving control of honeysuckle leaf spot. Additionally, VOCs produced by *Bacillus* only directly suppress the hyphal growth of pathogenic fungi but also indirectly alter the distribution of microorganisms by improving the soil environment. For instance, Sudakov et al. [89] reported that the VOCs produced by *Bacillus* could significantly reshape the rhizosphere microbial community structure, reduce the soil humus content, increase the soil pH, and upregulate key metabolic pathways, such as phenylpropanoid biosynthesis, carbohydrate metabolism, and hormone signal transduction in plants, thereby regulating plant growth at the molecular level. Ye et al. [90] reported that volatile organic compounds released by *B. velezensis* FZB42 can remodel the root architecture of *Arabidopsis thaliana* and significantly promote primary root elongation. These VOCs also act through an auxin-dependent pathway, upregulating the expression of auxin synthesis genes and efflux carriers (PIN1/PIN2), enhancing polar transport, and relying on the core ARF7/ARF19 signaling pathway. In the context of salt stress, researchers [91] reported that volatile organic compounds released by *B. velezensis* SQR9 can regulate salt tolerance defence through two pathways: Scavenging ROS, increasing antioxidant enzyme activities, and maintaining ion homeostasis (high K^+^/Na^+^). Its core active component, which can mimic its salt-tolerant and growth-promoting effects, significantly enhances the salt tolerance of *Arabidopsis thaliana*. During postharvest storage, susceptibility to fungal infection increases because of moisture, but traditional chemical fungicides and sulfur fumigation pose safety risks. Researchers [92] have reported that high concentrations of the VOCs released by *Bacillus thuringiensis* G-5 can efficiently inhibit the activity of two pathogenic fungi. Among these compounds, 3-hepten-2-one was identified as the main antifungal component, which exerts its antifungal effects by disrupting the integrity of the pathogen cell membrane, causing leakage of cellular contents, and inhibiting ergosterol synthesis. VOCs produced by *Bacillus* sp. LNXM12 significantly inhibit postharvest pathogens. Infection rates on tomato and strawberry leaves and fruits were markedly reduced. When fungal mycelia were exposed to these VOCs, their morphology changed, resulting in damage, irregularity, lysis, and dark blue staining [93]. Moreover, *Bacillus* VOCs can achieve biocontrol effects either by directly inhibiting pathogens or by indirectly inducing plant resistance. Khan et al. [94] reported that compared with compounds from incompatible combinations or single strains, VOCs produced by a compatible *Bacillus* strain combination (LNXM12+LLTC93) suppressed the growth of *Botrytis cinerea*, the causal agent of tomato grey mold, more effectively. These VOCs induce reactive oxygen species production in fungal hyphae, causing cell structure distortion and shrinkage, while activating ISR in tomato plants and upregulating the expression of defence-related genes such as *PR1*, *PR3*, and *PAL*. In summary, *Bacillus* VOCs have broad application prospects in agricultural production, and most are biodegradable and do not cause environmental pollution. Table 2 summarizes the representative antifungal VOCs produced by different *Bacillus* strains, along with their target pathogens and host plants.

**Table 2.** Antifungal volatile organic compounds (VOCs) produced by *Bacillus* spp.

Antifungal VOCs	*Bacillus* Strain	Target pathogen	Ref.
2-octanol, acetic acid, 2-methylbutanoic acid, butanoic acid, isobutyric acid, propanoic acid	*Bacillus velezensis* P2-1	*Botryosphaeria dothidea*	[95]
Isovaleric acid, 2-methylbutanoic acid, benzaldehyde, trimethylamine, 2-octanone, 2,5-dimethylpyrazine	*Bacillus velezensis* HC-10	*Alternaria alternata*	[88]
2,4-di-tert-butylphenol	*Bacillus subtilis* 0618A	*Sclerotium rolfsii*	[96]
2-methylbutanoic acid, 5-methyl-2-hexanone, 2,3-hexanedione, 2-ethyl-1-hexanol, 6-methyl-2-heptanone	*Bacillus subtilis* A3	*Fusarium graminearum*	[97]
Ethyloctynol, 3-methyl-2-pentanone (3M2P), 1,3-butadiene, DMF, squalene	*Bacillus sp*. LNXM12	*Botrytis cinerea*	[93]
Acetoin, benzaldehyde, 2,3-butanedione, 2(3H)-furanone, 5-hexylidihydro-, 2,6-dimethylpyrazine	*Bacillus velezensis* BF01	*Botrytis cinerea, Fusarium oxysporum, Colletotrichum gloeosporioides*	[98]
2,4-hexadiene	*Bacillus velezensis* ITCE1	*Fusarium brachygibbosum, Penicillium* spp.	[99]
3-methylpentanoic acid, 1-octen-3-ol, 2,3-dihydrobenzofuran, 6-methyl-2-heptanone	*Bacillus safensis* N12B	*Pyricularia oryzae*	[100]
3-methylvaleric acid	*Bacillus toyonensis* D-6	*Penicillium crustosum, Alternaria alternata, Arthrinium phaeospermum*	[101]
3-hepten-2-one	*Bacillus thuringiensis* G-5	*Fusarium acuminatum, Penicillium oxalicum*	[92]
Ethyl 2-methylbutanoate, tridecane	*Bacillus paralicheniformis* 2-12	*Fusarium oxysporum*	[102]
2-dodecanone, 2-undecanone, 2-heptanone	*Bacillus velezensis* LT1	*Sclerotium rolfsii*	[103]
1-propanol, 2-methyl-1-undecanol	*Bacillus spp*. (LNXM12 + LLTC93 compatible combination)	*Botrytis cinerea*	[94]
5-nonylamine, dimethyl disulfide	*Bacillus amyloliquefaciens* HK17	*Sclerotium rolfsii*	[104]
Pentanoic acid, 3-methylbutanoic acid, ethyl heptanoate	*Bacillus siamensis* NEAU-ZGX24	*Botrytis cinerea*	[105]

#### 3.1.3. Hydrolytic enzymes

*Bacillus* species secrete a variety of hydrolytic enzymes that synergistically disrupt the fungal cell wall to achieve control of plant-pathogenic fungi. Chitinases and β-1,3-glucanases hydrolyze the core cell wall skeletons of chitin and glucan; proteases degrade glycoproteins in the wall and destroy the extracellular enzymes and toxins of the pathogen; and cellulases and pectinases further degrade supporting structures such as cellulose and pectin. This concerted action leads to hyphal disintegration and, together with secondary metabolites such as antibiotics, contributes to broad-spectrum antifungal activity [106].

Chitinases mainly inhibit fungal growth directly by hydrolyzing the chitin component of the pathogenic fungal cell wall, disrupting cell wall integrity, and causing hyphal malformation, rupture, and impaired spore germination [107],[108]. Furthermore, chitinases can degrade the eggshells and larval cuticles of nematodes and synergistically enhance their insecticidal effect when combined with the crystal toxins of *Bacillus thuringiensis* [109]. Moreover, the chitin oligosaccharides generated by chitinase hydrolysis can act as plant immune elicitors, inducing systemic defence responses in plants, including the activation of pathogenesis-related (PR) proteins and phytoalexin synthesis, thereby indirectly strengthening plant resistance to pathogens [110]. Proteases produced by *Bacillus* species can inhibit spore germination and hyphal growth by degrading structural proteins and glycoproteins in the fungal cell wall, thereby compromising cell wall integrity. Specifically, proteases hydrolyze the protein matrix of the fungal cell wall into polypeptides and amino acids, acting synergistically with chitinases and β-1,3-glucanases to cause hyphal swelling, malformation, cell wall perforation, and lysis [107]. Additionally, proteases can degrade proteins associated with pathogen infection, directly attenuating their pathogenicity [111]. Cellulases can hydrolyze cellulose in the cell walls of oomycetes, leading to cell lysis, deformation, and leakage of cellular contents, thereby inhibiting hyphal growth and spore germination [112].

On the basis of existing research, the antifungal mechanisms of *Bacillus* are by no means attributable to a single hydrolytic enzyme. Khattak et al. [113] isolated a strain of *B. velezensis* from the eggplant rhizosphere that destroyed the cell wall and plasma membrane of *Verticillium dahliae*, inhibited spore germination, and caused hyphal malformation through the production of chitinase and cellulase, among others. Choub et al. [114] utilized *B. subtilis* CV21, which produces cell wall-degrading enzymes such as chitinase and protease, to disrupt the cell wall of the pathogenic fungus *Alternaria alternata*, leading to hyphal swelling, rupture, and impaired spore germination, thereby achieving control of plant-pathogenic fungi. Xue et al. [115] discovered that *B. velezensis* K01 could produce multiple hydrolytic enzymes, including protease and cellulase, and exhibited broad-spectrum antagonistic activity against 12 plant pathogenic fungi and 5 pathogenic bacteria *in vitro*. Genomic analysis further revealed 208 carbohydrate-active enzyme (CAZyme) genes, and these hydrolytic enzymes constitute important bases for its antimicrobial and growth-promoting effects.

### 3.2. Siderophores and induced systemic resistance

Siderophores secreted by *Bacillus* species have an extremely high affinity for Fe^3+^. These siderophores can chelate trace amounts of soluble iron in the soil or plant rhizosphere environment under iron-limited conditions, such as those in the rhizosphere, thereby significantly reducing the concentration of iron available to fungi. Since iron is an essential element for fungal growth and pathogenicity, iron deficiency inhibits fungal growth and development [116]. Liu et al. [117] reported that *B. velezensis* YL2021 produced catecholate-type siderophores, including bacillibactin, under low-iron conditions. These siderophores exhibited broad-spectrum antimicrobial activity, and *in vitro* inhibition assays and pot experiments demonstrated their effectiveness in controlling rice sheath blight and rice blast. The interaction of certain bacteria with plant roots can lead to plant resistance to certain pathogenic bacteria, fungi, and viruses. This phenomenon is referred to as ISR [118]. Furthermore, siderophores secreted by some *Bacillus* species can also be recognized by plant roots as elicitors. This triggers ISR in the plant, priming the plant to enter a defence-ready state so that upon subsequent fungal attack, the plant can more rapidly thicken its cell walls and accumulate antimicrobial substances, indirectly enhancing resistance to fungal diseases. Zhu et al. [119] reported that endophytic *B. velezensis* could induce ISR in *Atractylodes macrocephala* by activating the jasmonic acid/ethylene (JA/ET) signaling pathway while triggering systemic acquired resistance through synergistic action with the salicylic acid pathway. Hu et al. [120] discovered that the genome of an endophytic *Paenibacillus peoriae* strain harbored ISR-associated genes, such as those for 2,3-butanediol, methanethiol, and isoprene. Concurrently, transcriptome analysis revealed that treatment with this strain upregulated the expression of NPR1, a key regulatory gene in salicylic acid signalling, and increased the activity of plant–pathogen interactions and MAPK signaling pathways.

### 3.3. Nutrient and spatial competition

Nutrient and spatial competition constitute key components of biological control by *Bacillus* species. *Bacillus* can rapidly and firmly colonize ecological niches such as the root surface, vascular bundles, or wound sites of plants, forming dense biofilms that block contact between fungal hyphae and host tissues [121]. Extracellular polymeric substances (EPSs) produced by bacteria improve water retention and uptake in the rhizosphere region through biofilm formation and are highly beneficial for stabilizing soil aggregates and regulating nutrient and organic carbon sources [122]. Furthermore, *Bacillus* in the rhizosphere competes with pathogenic fungi for key nutrients such as iron by secreting substances such as siderophores, thereby limiting fungal growth. The extracellular matrix physically encapsulates and agglutinates fungal hyphae and spores, interfering with their development and dispersal, thus achieving dual competitive inhibition of space and nutrients [123]. Liu et al. [124] reported that *Bacillus halotolerans* 105 competed with pathogens for nutrients such as iron through siderophore production while efficiently colonizing the root surface by utilizing intact chemotaxis and biofilm formation gene modules and seizing nutrients and space, thereby achieving spatial and nutrient competitive inhibition of various plant pathogenic fungi under salt stress.

In summary, the biocontrol efficacy of *Bacillus* species against plant pathogenic fungi relies on the direct secretion of antimicrobial substances and indirect competition for nutrients and space, which together prime the plant to mount a more rapid and robust defence response upon pathogen attack. These mechanisms do not operate in isolation; rather, antibiosis, nutrient and spatial competition, and the induction of plant immunity interact and act synergistically, resulting in the construction of a multilayered biocontrol defence system that ensures that *Bacillus*-based agents consistently exert disease-suppressive effects across cropping systems.

## 4. From single strains to synthetic consortia

The biocontrol mechanism of *Bacillus* includes multiple pathways, such as competition between nutritional and spatial sites, the production of antimicrobial substances, and the induction of plant systemic resistance. However, a biocontrol strategy that relies on a single strain has limitations in practical field applications. Under complex soil microecology conditions and strong fluctuations in temperature and humidity, the colonization ability of soil easily declines sharply. Moreover, the types of antibacterial compounds produced by single strains are limited, and the antibacterial spectrum is relatively narrow; thus, preventing and controlling the disease caused by multiple pathogens at the same time is difficult. To overcome the above limitations of a single strain, two or more microorganisms with complementary or synergistic functions can be made into biocontrol agents, and the biocontrol effect on plant-derived fungi can be enhanced by a synergistic multistrain combination strategy [125].

Compared with a single strain, a multistrain combination can result in more stable niche coverage in the rhizosphere [126]. For example, Zhou et al. [127] constructed a minimal synthetic consortium (SynCom) with *Trichoderma harzianum* T19 and *Bacillus firmus* PM16, which showed increased ecological adaptability in the wheat rhizosphere. Studies have shown that SynCom can stably colonize plants under the pressure of pathogens, significantly increasing the enzyme activity of rhizosphere soil and effectively overcoming the problem of decreased colonization ability of a single strain under stress conditions. In addition, Qiao et al. [128] reported that SynCom composed of *Bacillus* and *Streptomyces* could be stably colonized in the apple rhizosphere for 84 days and was maintained at a concentration of 10^6^ CFU/g, which was significantly better than the survival rate of a single strain. These findings indicate that multistrain combinations can significantly increase the persistence of exogenous beneficial microorganisms in complex rhizosphere environments through complementary niche occupation and metabolic synergy. Sun et al. [129] reported that the beneficial rhizosphere bacterium *B. velezensis* SQR9 could significantly increase the abundance of members of another biocontrol genus, Lysobacter, upon infection by the pathogenic fungus *Fusarium oxysporum*. Further experiments revealed that *Bacillus* and *Lysobacter* formed synergistic biofilms through metabolic cross-feeding and upregulation of the production of antifungal metabolites (such as bacillomycin D and HSAF). In cucumber pot experiments, compared with single-strain treatments, the two-species consortium consisting of *B. velezensis* SQR9 and *L. enzymogenes* XL8 significantly reduced the disease index of *Fusarium* wilt and decreased pathogen abundance. These findings indicate that synthetic microbial consortia can effectively enrich beneficial bacteria.

The combination of multiple strains can significantly increase the types and yields of antibacterial compounds, thereby enhancing the inhibitory ability against a variety of pathogenic bacteria. Compared with a single strain, different members of SynCom can produce antimicrobial metabolites with different targets, which not only improve the control effect on major pathogens but also effectively inhibit the coinfection of multiple pathogens and delay the development of pathogen resistance. Song et al. [130] constructed SynCom composed of eight *Bacillus* strains and achieved synergistic production of various metabolites, such as IAA, siderophores, lipopeptides, and polyketides, through metabolic division mechanisms. The control effect of SynCom on rice sheath blight was 70.28%, and the growth of the rice was significantly promoted. In addition, by addressing the severe damage caused by *Ralstonia solanacearum* to tobacco through bacterial wilt and the limited effectiveness of traditional control methods, Liu et al. [131] isolated bacteria from the rhizosphere soil of healthy tobacco plants and constructed a chemotactic synthetic community (SynCom) consisting of two *Pseudomonas* strains and one *Bacillus* strain. By screening prebiotics (such as vitamin C and propionic acid) from root exudates to increase the antagonistic ability of SynCom and optimizing the proportion of organic fertilizer, they developed a bioorganic fertilizer. This approach not only controlled bacterial wilt but also increased tobacco yield.

SynCom can promote the improvement of community stability through synergistic interactions between its members. The combination of multiple strains not only directly acts on pathogens but also indirectly inhibits the occurrence of diseases by reshaping the co-occurrence network of rhizosphere microbial communities. Ali et al. [132] isolated a *Bacillus*-dominated flora from wax gourd leaves for the construction of SynComs. After inoculation with SynCom, the abundance of beneficial bacteria in the phyllosphere microbiome of wax gourd increased significantly, the complexity of the microbial co-occurrence network increased, and the α diversity index increased. These findings indicate that the mutual cooperation of beneficial bacteria can improve the stability and disease resistance of the community and promote plant growth by reshaping the microbial community structure and enhancing network connectivity. Bashizi et al. [133] isolated five bacterial strains with plant growth-promoting and antagonistic functions from the rhizosphere of healthy pepper plants (*Bacillus* sp., *Flavobacterium anhuiense*, *Cytobacillus firmus*, *Streptomyces roseicolaratus*, and *Pseudomonas frederiksbergensis*) and constructed a synthetic microbial community. SynCom treatment reshaped the rhizosphere microbial community, enriching beneficial bacteria (e.g., *Bacillus*) and fungi (e.g., *Fusicolla*, Trichoderma), and enhanced functional pathways related to nitrogen metabolism and secondary metabolite biosynthesis, thereby synergistically improving pepper resistance against *Phytophthora capsici*. It significantly reduced the severity of *Phytophthora* blight; promoted plant growth (increasing plant height, root length, biomass, chlorophyll content, etc.); and increased the levels of nitrate‑nitrogen and potassium in the rhizosphere soil.

In summary, SynComs can overcome the key limitations of single-strain biocontrol agents, including poor colonization ability under environmental stress, narrow antimicrobial spectra, and limited disease suppression. By occupying rhizosphere ecological niches, diversifying the types of antimicrobial metabolites produced, and reshaping the structure of native microbial communities, SynComs can enhance community network stability and plant disease resistance. These complementary and synergistic effects make synthetic microbial communities a promising and sustainable strategy for the management of soil-borne fungal and bacterial diseases in agriculture.

## 5. Application in the biocontrol of phytopathogenic fungi by *Bacillus* species

The screening and antagonistic mechanism of the biocontrol of *Bacillus* and the strategy evolution from a single strain to synthetic flora have been briefly described. In actual production, the value of *Bacillus* is far greater than inhibiting pathogenic fungi. It promotes growth, dissolves phosphorus, fixes nitrogen and so forth. It can promote the healthy growth of crops while preventing disease. As shown in Figure 2, in this section, we briefly describe the application of *Bacillus* in the biological control of plant pathogenic fungi in four scenarios: food crops, legume crops, fruit trees, medicinal plants, and extreme environments.

**Figure 2.**
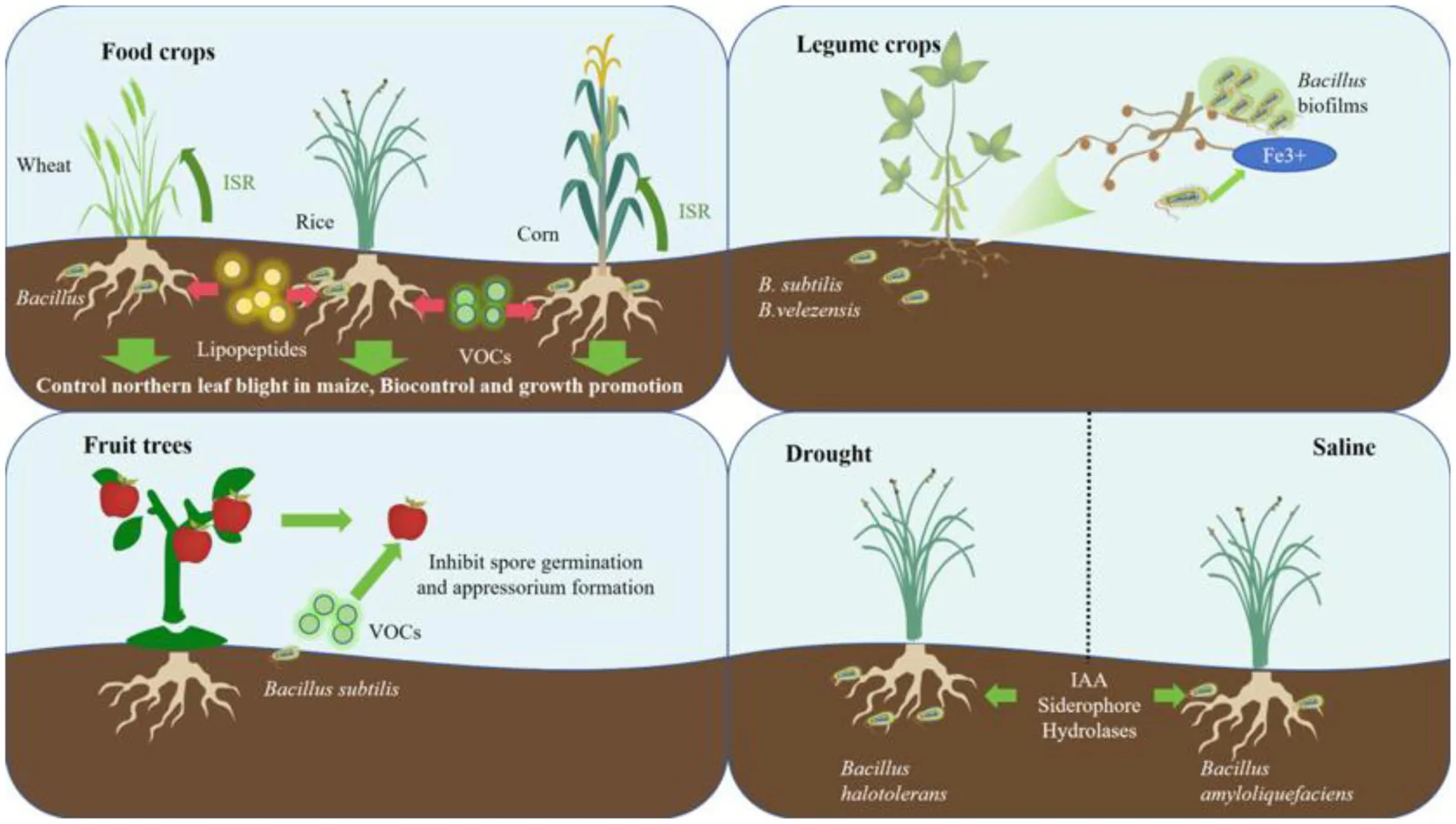
Application in the biocontrol of phytopathogenic fungi by *Bacillus* species [(A) Food crops: Root-colonizing *Bacillus* releases lipopeptides and VOCs to trigger ISR, promote growth of wheat, rice and maize, and control northern leaf blight. (B) Legume crops: *Bacillus subtilis* and *Bacillus velezensis* form biofilms and secrete siderophores to chelate Fe^3+^, improving iron nutrition. (C) Fruit trees: *B*. *subtilis* VOCs inhibit fungal spore germination and appressorium formation to protect against fungal diseases. (D) Abiotic stress: Stress-tolerant *Bacillus* strains synthesize IAA, siderophores, and hydrolases; *B. halotolerans* mitigates drought damage; and *B. amyloliquefaciens* enhances salt tolerance.

### 5.1. Disease control in food crops

Food crop diseases seriously threaten global food security. Cereal crops such as wheat, rice, and corn are perennially threatened by fungal diseases such as scab, corn leaf blight, leaf blight, and rice blast [134]–[136], which can lead to no harvest in severe cases. The use of *Bacillus* and its active substances to inhibit pathogens through the secretion of antifungal substances, nutrient competition, and the stimulation of plant immunity provides a new method for the green control of fungal diseases in food crops. For example, *B. velezensis* JCK-7158 produces antifungal substances such as iturin A, surfactins, and volatile organic compounds that directly inhibit *Fusarium* head blight. Field application revealed that a 1000-fold diluted suspension formulation could significantly reduce the disease index of rice scab and the content of nivalenol toxin and increase yield [137]. *Bacillus tequilensis* enhances the resistance of plants to powdery mildew by inducing systemic acquired resistance in wheat and upregulating the expression of pathogenesis-related protein genes, various defence-related genes, and related enzyme activities. At the effective concentration, the *in vitro* inhibition rate of wheat powdery mildew ranged from 90.91% to 100%, and the field control effect ranged from 83.21% to 100% [138]. *B. velezensis* EM-A8 can effectively control northern leaf blight in maize. In a field experiment, the number of susceptible leaves was reduced by 25%–50%. Moreover, bacteria increased the dry biomass of maize by 30% by synthesizing plant hormones such as salicylic acid, auxin, and cytokinin and had dual functions in terms of biocontrol and growth promotion [139]. As a biocontrol agent, *Bacillus* can effectively prevent and control major fungal diseases of food crops by secreting antimicrobial lipopeptides and volatile substances, inducing systemic resistance, and synthesizing plant hormones. Moreover, it can increase yield and promote growth, providing a feasible and efficient new way to achieve green prevention and control of fungal diseases of food crops.

### 5.2. Disease control in legume crops

Legume crops, especially faba bean, mainly face root rot, which is caused by a variety of pathogens, including *Fusarium, Rhizoctonia*, and *Pythium*. This disease can cause 5%–30% yield loss [140]. When *B. subtilis* alone or mixed with *Pseudomonas* and *Rhizobium* is used to treat seeds, the disease index of *Fusarium* root rot can be reduced by 50%–62%, the plant height can be significantly increased, and the combined treatment has a synergistic effect [141]. Sendi et al. [142] reported that a variety of *Bacillus* strains screened from rhizosphere and endophytic bacteria strongly inhibited root rot, and the inhibition rate ranged from 50% to 71% *in vitro*. A greenhouse pot experiment also revealed that the severity of root rot significantly decreased. The combined use of *Bacillus* and *Rhizobium* not only reduces the incidence of root rot but also significantly improves the stability of the rhizosphere microbial network and increase plant biomass, resulting in a synergistic effect [143].

### 5.3. Disease control of fruit trees and medicinal plants

The loss of fruits after harvest caused by plant pathogenic fungi is estimated to account for more than 50% of the total yield of agricultural fruits [144]–[146]. The common diseases are brown rot caused by *Monilinia*; blue mold and green mold caused by *Penicillium*; anthracnose caused by *Colletotrichum*; grey mold caused by *Botrytis cinerea*; and *Alternaria* rot caused by *Alternaria*. [147],[148]. The use of metabolites produced by bacteria to inhibit plant pathogens can improve the efficiency of controlling fruit diseases [112],[149]. Heo et al. [150] isolated a strain of *B. subtilis* GYUN-2311 from the rhizosphere soil of an apple orchard. It effectively prevented and controlled anthracnose on apple and pepper fruits *in vitro* by producing volatile organic compounds, culture filtrates, and lyases to inhibit spore germination and appressorium formation of pathogenic bacteria, and the control effect was 70%–75%. Lou et al. [151] reported that *B. subtilis* Sjb3 had a broad-spectrum inhibitory effect of 39.34%–47.50% on seven plant pathogenic fungi, such as rose anthracnose, black spot, and apple ring rot. *In vitro* inoculation experiments further revealed that the Sjb3 strain could significantly reduce lesion expansion on rose leaves and apple fruits.

Medicinal plants are susceptible to a variety of diseases, which may severely affect their growth, yield and medicinal value. The control of *Bacillus* not only provides fungal control for the cultivation of medicinal plants but also provides a sustainable method that considers yield, quality, and the environment [152]. Zhu et al. [119] isolated the endophyte *Bacillus velezensis* Amzn015 from healthy *Atractylodes macrocephala*. The strain showed broad-spectrum antibacterial activity against four pathogenic fungi and promoted plant growth. Further pot experiments revealed that Amzn015 significantly reduced the incidence of *A. macrocephala* root rot, with a relative control effect of 66.2%, and promoted growth and disease prevention. Moreover, medicinal plants contain unique bioactive metabolites and microbial communities, which can provide a rich and diverse beneficial microbial community [153]. Patil et al. [154] isolated and screened *Bacillus* strains from the rhizosphere soil of medicinal plants. These strains can stimulate systemic resistance and significantly reduce the incidence of sheath blight by producing antibiotics and inducing defence enzyme activity. Field experiments confirmed that *Bacillus toyonensis* treatment could reduce the incidence of rice sheath blight to 12.88%–17.37% and significantly increase yield.

### 5.4. Climate Change Constraints and Coping Strategies

The stability of field efficacy of biocontrol agents is largely constrained by fluctuations in climatic conditions. Abiotic factors such as temperature, humidity, and light directly or indirectly alter the ability of biocontrol agents to suppress diseases by influencing their survival, colonization, growth, and metabolite secretion. Most biocontrol bacteria and fungi have an optimal temperature range for growth; deviations from this range can inhibit spore germination, mycelial growth, and the synthesis of antimicrobial compounds. For example, at 23 °C, a *Bacillus*-based biocontrol product showed significant efficacy against carrot disease, but under high temperatures, its direct antifungal activity and induced systemic resistance (ISR) were affected to varying degrees [155]. Studies have revealed that although high-humidity weather or increased dew points can significantly reduce the mortality of *Bacillus*, the accompanying rain wash-off and the decline in viable cell concentration per unit area still limit the biocontrol effect [156].

The impacts of climate change on microbial activity, biodiversity, and plant growth and productivity are intensifying aridification in many ecosystems, while different microorganisms vary in their sensitivity to high temperatures and drought [157]. These changes have significantly affected global agricultural production. In addition, factors such as rising temperatures, elevated tropospheric ozone concentrations, acid rain, and increased atmospheric CO_2_ levels can place plants under long-term stress, thereby reducing their resistance to pathogens [158]. Climate change driven by warming and shifts in humidity may further promote the emergence of new pathogenic strains and reduce the abundance of other microbial groups. Studies have revealed that long-term warming has led to a significant increase in the relative abundance of fungi in Arctic tundra soils, while long-term CO_2_ exposure and warming adversely affect the functions of soil fungal communities in paddy fields, threatening crop health and soil functions [159]. The thermal expansion of warm seawater and the melting of ice sheets and glaciers are intensifying salinity stress for organisms worldwide. Elevated salinity reduces soil microbial biomass and disrupts enzyme activity by affecting enzyme structure. Researchers have found that *B*. *siamensis* BW can maintain plant health and growth under saline–alkali stress by producing antioxidants and stimulating plant growth, thereby alleviating oxidative damage [160]. Since the 1950s, global flood risk has been continuously increasing. Flooding causes soil hypoxia and kills or injures terrestrial plants through the accumulation of toxic substances, cytoplasmic acidification, and disease. In contrast, plant growth-promoting bacteria of the genus *Bacillus* can protect plants from flooding stress by reducing ethylene accumulation [157].

Compared with other biocontrol agents, biocontrol microorganisms such as *Bacillus* spp. are of great research value because they can form highly stress-resistant spores. In recent years, researchers have gradually turned their attention to isolating stress-tolerant biocontrol strains from extreme habitats and exploring their application potential under extreme agricultural conditions. Zhao et al. [161] isolated *Bacillus* sp. TR005 and other strains from medicinal plants in arid areas of Xinjiang, which strongly inhibited four pathogenic fungi and strongly promoted siderophore production, indicating that in extremely arid environments, *Bacillus* endophytes are resistant to stress, providing valuable strain resources for plant disease prevention and control in arid areas. Moreover, the *Bacillus* strains obtained by Muñoz-Torres et al. [162] in the Atacama Desert showed a variety of plant growth-promoting characteristics, including phosphate solubilization, nitrogen fixation, and siderophore production, and could secrete proteases, lipases, and other hydrolases, which helped improve soil nutrient availability under extremely poor conditions, indicating the potential of *Bacillus* as a biological fertilizer suitable for arid saline-alkaline environments. Table 3 lists the stress-tolerant *Bacillus* strains and their biocontrol potential under extreme environments, including salt, drought, and heat stress.

**Table 3.** Biocontrol potential in extreme environments.

*Bacillus* Strain	Tolerated Environment	Biocontrol Fungi	Plant Growth Promotion	Ref.
*Bacillus amyloliquefaciens* VFS2	Salt stress	*Fusarium equiseti, Fusarium. oxysporum*	IAA, siderophores	[163]
*Bacillus velezensis*, *Bacillus amyloliquefaciens*	Drought stress	*Fusarium graminearum, Fusarium culmorum*	IAA, ACC deaminase, siderophores	[164]
*Bacillus*, *Pantoea*	Salt- and heat-tolerant	*Botrytis cinerea*, *Monilinia fructicola*	IAA, siderophores, solubilizes phosphate, fixes nitrogen	[162]
*Bacillus*	salt-tolerant	*Fusarium proliferatum*		[165]
*Klebsiella pneumoniae*, *Bacillus amyloliquefaciens*, *Bacillus subtilis* MEBAphL4	Salt-tolerant	*Fusarium solani*, *Talaromyces amestolkiae*, *Cladosporium tenuissimum*	ACC deaminase, solubilizes phosphate, produces ammonia, fixes nitrogen, extracellular enzymes	[166]
*Bacillus velezensis* NH-13-5	Salt-tolerant, pH-tolerant	*Botrytis cinerea*		[167]
*Bacillus halotolerans Strain 105*	Salt-tolerant	*Fusarium oxysporum, Fusarium moniliforme*, *Fusarium solani*, *Fusarium proliferatum*	IAA, protease	[168]

### 5.5. Commercial *Bacillus* biocontrol agents

Microorganisms of the genus *Bacillus* are the most widely used group of biocontrol bacteria. In terms of their action mechanisms, the roles that these widely applied commercial microbial agents play lie in biodiversity, soil carbon and nitrogen cycling, microbial community assembly, and pollution bioremediation [169].

When commercial formulations are applied to plants, their formulation processes often face several problems in field application: The major physical forms of the formulations, liquid, solid, or slurry, make it difficult to protect the microbial strains from abiotic stresses such as temperature, humidity, salinity, UV radiation, and pH during application. Studies have shown that encapsulation with polymers is an effective alternative strategy to increase microbial survival, as it can protect microorganisms from environmental conditions. Commonly used adsorbent carriers include peat, coal, clay, talc, vermiculite, charcoal, cellulose, sawdust, wheat bran, alginate beads, rice husk, polyacrylamide gel, gypsum, and silica [170].

The role of microbial agents is not limited to the inhibition of plant-pathogenic fungi; they can also enhance the activities of soil enzymes during crop growth [171]. When microbial agents are applied to the soil, they promote the rapid growth of soil microorganisms, promote the rapid growth of soil microorganisms. This further influences the agronomic traits of food crops. Table 4 summarizes the *Bacillus* biocontrol agents reported in recent years, detailing their carrier materials, formulation types, and functional roles in agricultural applications.

**Table 4.** *Bacillus* biocontrol agents.

Strain	Carrier	Formulation Type	Function	Ref.
*Bacillus subtilis*	Rice straw biochar	Soil amendment	Improving microbial community structure, enzyme activity, and microbial biomass carbon/nitrogen	[172]
*Bacillus subtilis*	Chitosan gel beads	Gel beads	Long-lasting, slow-release biocontrol against *Fusarium avenaceum* and *Rhizoctonia solani*	[173]
*Bacillus halophilus* BH-8	Coal gangue	Composite microbial agent	Increasing soil available nitrogen, available phosphorus, available potassium, and organic matter	[174]
*Bacillus subtilis* LFB1249	Maltodextrin	Wettable powder	Inhibitory effect on *Rhizoctonia solani* and *Fusarium graminearum*, with a storage period of 24 months	[175]
*Bacillus subtilis* BS191	KOH/urea co-modified walnut pericarp biochar	Biochar-based microbial agent	Control of cotton verticillium wilt	[176]
*Bacillus subtilis*		Wettable powder	Control of tobacco target spot disease caused by *Rhizoctonia solani*	[177]
*Bacillus subtilis* ZF71	Sodium alginate/pectin hydrogel	Seed coating	Control of cucumber Fusarium root rot caused by *Fusarium solani*	[178]
*Bacillus megaterium*	Low-temperature carbonized/activated lignin-based porous carbon	Phosphate-solubilizing bacterial agent	Enhance soil phosphorus utilization	[179]
*Bacillus velezensis* P15		Fermentation broth	Control of postharvest diseases of peach	[180]

## 6. Conclusions and future perspectives

*Bacillus* spp. have become important microbial resources for the biological control of plant pathogenic fungi because of their broad-spectrum antifungal activity, unique spore resistance, and biocontrol mechanisms. In this paper, the screening of biocontrol strains of *Bacillus*, the biocontrol mechanism, the construction strategy of a multistrain synthetic consortium, and the progress of field application are reviewed. These studies have shown that *Bacillus* can directly inhibit pathogenic fungi by secreting lipopeptides, volatile organic compounds, and hydrolases; *Bacillus* can also compete for nutrition and space through siderophores to block the colonization of pathogens. Furthermore, it induces systemic resistance in plants and activates the host defence response to achieve biological control of plant pathogenic fungi. In addition, *Bacillus* also has growth-promoting effects, such as nitrogen fixation, phosphorus solubilization, and the production of plant hormones, which directly promote plant nutrient absorption and growth and development and achieve synergistic effects of disease prevention and increased yield. *Bacillus*-mediated biological control is also shifting from “single strain–single mechanism” to the synergistic development paradigm of “synthetic flora–system regulation”, providing an environmentally friendly, cost-effective, and sustainable alternative strategy for treating crop fungal diseases. However, there are many problems associated with the commercial application of *Bacillus* biocontrol agents, which may be developed in the following directions in the future: (1) In the field soil environment, its colonization efficiency and duration are often affected by factors such as pH, temperature and humidity, as well as competition among indigenous microorganisms. In the future, it may be possible to improve the field colonization ability of this species by developing carrier materials. (2) At present, the construction of SynComs mostly depend on empirical screening. In the future, machine learning can be used to standardize the minimum functional flora SynCom. (3) With the intensification of climate change, strong stress-tolerant strains should be excavated from extreme habitats such as deserts, hot springs and salt lakes, and multifunctional strains should be developed by combining adaptive evolution and CRISPR breeding technology [181]. (4) With the development of smart agriculture, *Bacillus* biocontrol agents have been combined with smart agricultural technologies such as precision irrigation and drone application, and the biocontrol mechanism has been combined with traditional fungal prevention and control measures such as intercropping to enhance fungal prevention and control capabilities and achieve intelligent disease management [182].

## Use of AI tools declaration

The authors state that they have not utilized Artificial Intelligence (AI) tools when creating this article.
